# Machine learning workflow to enhance predictions of Adverse Drug Reactions (ADRs) through drug-gene interactions: application to drugs for cutaneous diseases

**DOI:** 10.1038/s41598-017-03914-3

**Published:** 2017-06-16

**Authors:** Kalpana Raja, Matthew Patrick, James T. Elder, Lam C. Tsoi

**Affiliations:** 1 Department of Dermatology, University of Michigan Medical School, Ann Arbor, MI USA; 2 Department of Computational Medicine & Bioinformatics, University of Michigan Medical School, Ann Arbor, MI USA; 3 Department of Biostatistics, University of Michigan, Ann Arbor, MI USA

## Abstract

Adverse drug reactions (ADRs) pose critical public health issues, affecting over 6% of hospitalized patients. While knowledge of potential drug-drug interactions (DDI) is necessary to prevent ADR, the rapid pace of drug discovery makes it challenging to maintain a strong insight into DDIs. In this study, we present a novel literature-mining framework for enhancing the predictions of DDIs and ADR types by integrating drug-gene interactions (DGIs). The ADR types were adapted from a DDI corpus, including *i*) adverse effect; *ii*) effect at molecular level; *iii*) effect related to pharmacokinetics; and *iv*) DDIs without known ADRs. By using random forest classifier our approach achieves an F-score of 0.87 across the ADRs classification using only the DDI features. We then enhanced the performance of the classifier by including DGIs (F-score = 0.90), and applied the classification model trained with the DDI corpus to identify the drugs that might interact with the drugs for cutaneous diseases. We successfully predict previously known ADRs for drugs prescribed to cutaneous diseases, and are also able to identify promising new ADRs.

## Introduction

Adverse drug reactions (ADRs) are harmful reactions, resulting from the intake of one or more drug(s) and pose significant public health issues. In the USA, ADRs affect >6% of hospitalized patients, with 0.3% being fatal incidences^1^. ADRs may occur due to prolonged administration of a drug, or combined usage of two or more drugs^2^. ADRs occur significantly higher in hospitalized cases^3^, and ADRs have significant impact on different age groups: drug reactions and toxicity can be different for children than adults, and ADRs occur more frequently for elderly people than other age groups, due to a higher disease prevalence and incidence of multi-morbidities^4^. In addition to the clinical burden, ADRs also impose significant economic cost. In the United States, hospital admission due to ADRs costs >$100 billion and long-term care admissions cost >$30 billion^5^. While knowledge of potential drug-drug interactions (DDI) is necessary to prevent ADR, maintaining the information about DDIs is challenging with the rapid growth of drug discovery.

Biomedical literature is the main source of knowledge on DDIs and ADRs. The PubMed database contains close to 5,000 articles for DDIs and >7,000 articles for ADRs related to human as of Jan, 2017. DDIs and ADRs have received much interest from patient health care in recent years. This is evident from close to 70% of DDI articles and >50% of ADRs articles being published in the last ten years. Automated approaches have been developed for the recognition and extraction of DDIs or ADRs alone^6–8^, and there is an increasing interest in identifying ADRs caused by DDIs using text-mining approaches^9^. Since DDIs may occur when two drugs interact with the same gene^8^ or when one drug inhibits or induces the metabolic pathway of the other drug^10^, it has also been suggested that incorporating drug-gene interactions (DGIs) can enhance the prediction of DDIs^8^. In addition to DDIs and ADRs identification, DGIs are also used as features by approaches based on combinatorial therapy to identify new drug combinations for complex disorders^11^.

Recent approaches on ADR prediction utilize the fact that drugs with similar chemical structure or drugs interacting with the same protein targets may lead to ADRs^12^. In this study, we present a novel and robust literature-mining framework for enhancing the predictions of DDI-based ADRs by integrating DGIs. We employed machine learning models to learn syntactic and semantic information from the literature, and took advantage of DGIs to advance the identification of DDIs and the classification of ADR types. We used four ADR types described previously^9^: (*i*) adverse effect; (*ii*) effect at molecular level; (*iii*) effect related to pharmacokinetics; and (*iv*) drug interactions without known ADR. Specifically, we combined DGIs and their associations (i.e. acetylation, methylation, phosphorylation, etc.) to enhance the performance of classification. We showed that our integrative method could improve DDI prediction and ADR classification by incorporating DGIs. Finally, we applied our approach to predict drugs that can potentially interact with drugs prescribed for different cutaneous diseases. We successfully predicted previously known ADRs, and also identified promising new ADRs for drugs prescribed to cutaneous diseases.

## Results

**Figure 1:**
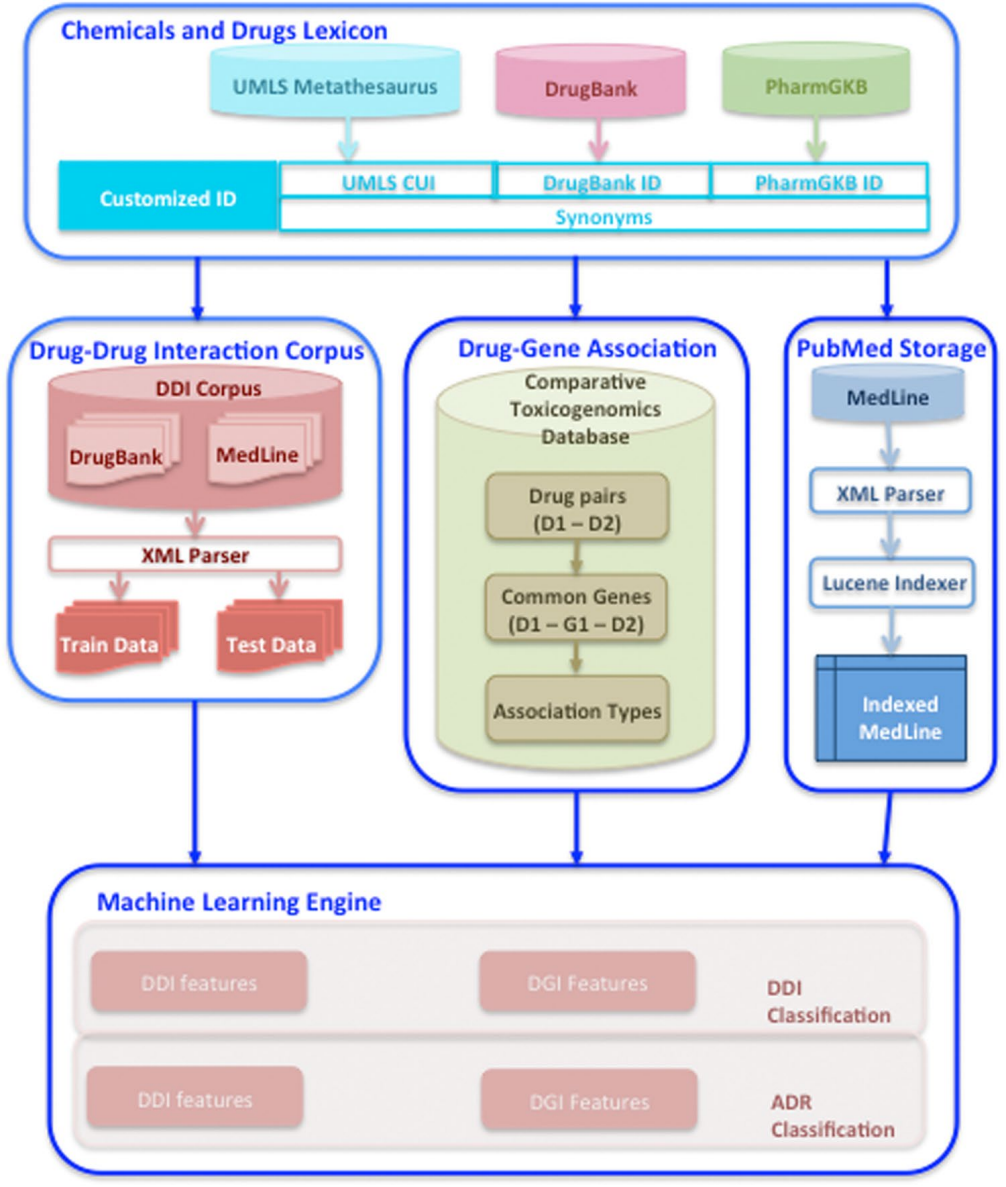
System architecture.

The features related to DDI and ADR types classification were selected from the drug-drug interaction (DDI) corpus, and DGIs were extracted from the Comparative Toxicogenomics Database (CTD)^13^. The literature-mining framework was developed to identify chemical/drug using a context-specific lexicon, and to represent the sentences from PubMed abstracts as feature vectors. The machine-learning workflow was developed to classify the feature vector using various classifiers namely Bayesian network, decision tree, random tree, random forest and k-nearest neighbors. The overall prediction workflow is shown in Fig. 1. In this section, we first report the performance of the chemicals and drugs lexicon on identifying drugs present in DDI corpus. Next, we evaluate the performance of various classifiers to predict DDIs and ADR types in DDI corpus using 10-fold cross validation technique. Finally, we apply the machine learning workflow on biomedical literature to predict DDIs and ADR types related to cutaneous diseases, and we discuss the findings and observations of our case study conducted on DDIs and ADR types related to psoriasis.

### Performance of lexicon on drug extraction

Table 1 shows the performance of using the chemicals and drugs lexicon (implemented as a concept dictionary with MedTagger) on identifying the drugs present in DDI corpus. The chemicals and drugs lexicon achieved an overall F-score (F) of 0.87 on training data and 0.78 (F) on test data. We performed a second evaluation to determine whether the entities belonging to the false positive (FP) category are in fact chemicals that are not annotated in the DDI corpus. We recalculated FP (annotated as FP1 in Table 1), precision (P1) and F-score (F1) by excluding the chemicals. Interestingly, the chemicals and drugs lexicon achieved an improved overall F-score (F1) of 0.94 on training data and 0.88 (F1) on test data. Manual checking of entities in false negative (FN) category revealed that the drugs are either brand names (e.g. Alfenta, Tysabri) or identified partially (e.g. warfarin instead of (R)-warfarin, magnesium hydroxide antacids instead of aluminum/magnesium hydroxide antacids), or the entities are drug classes (e.g. azole antifungals, quinolone antibiotics) that are not in the chemicals and drugs lexicon. Our lexicon includes chemicals in order to map Unified Medical Language System (UMLS) National Drug File – Reference Terminology (NDFRT)^14^ drugs that are actually chemicals (e.g. sulfur, salicylic acid, phenol).

**Table 1:** Performance of Chemicals and Drugs lexicon.

Dataset		True positive	False positive	False negative	FP1	Precision	Recall	F-score	P1	F1
Training (Cross validation)	DrugBank	11,051	2,060	932	373	0.84	0.92	0.88	0.97	0.94
	MedLine	1,372	484	335	6	0.74	0.80	0.77	1.00	0.89
	Overall	12,423	2,544	1,267	379	0.83	0.91	0.87	0.97	0.94
Test	DrugBank	279	61	17	46	0.82	0.94	0.88	0.86	0.90
	MedLine	288	191	58	34	0.60	0.83	0.70	0.89	0.86
	Overall	567	252	75	80	0.69	0.88	0.78	0.88	0.88

### Machine learning workflow on DDI/ADR types classification

We used DDI corpus training data to build the machine learning workflow on classifying DDIs and ADR types. First, we classified the sentences with potential DDI information, then we classified DDIs to four ADR types. The 10-fold cross validation on classifying the sentences with potential DDI information showed good performance when using DDI features alone or when employing both DDI with DGI features. The random forest classifier achieved the best performance of 0.80 F-score on DDI features alone and 0.81 F-score on DDI with DGI features (Table 2). Random forest also achieved the best performance among the five classifiers with macro (i.e. across the ADR types) average F-score of 0.87 for DDI features alone (Table 3), and improved to 0.90 when using both DDI and DGI features (Table 4). Though in a modest degree, the improvement is consistent across the different ADR type predictions: 0.89 vs. 0.91 for adverse effect; 0.87 vs. 0.91 for effect at molecular level; 0.81 vs. 0.84 for effect related to pharmacokinetics; and 0.90 vs. 0.92 for drug interactions without known ADR (Table 3 vs. Table 4). Importantly, we demonstrated that this observation is consistent among the other four classifiers: macro average F-score of 0.69 vs. 0.73 by Bayesian network, 0.86 vs. 0.89 by decision tree, 0.86 vs. 0.88 by random tree and 0.86 vs. 0.88 by k-nearest neighbors. Also, F-scores of individual ADR type predictions improved with DDI and DGI features when compared to DDI features in all four classifiers (Table 3 vs. Table 4).

**Table 2:** DDI Prediction comparison on DDI corpus training data.

Classifier	DDI Features			DDI and DGI Features			DGI Features		
	Precision	Recall	F-score	Precision	Recall	F-score	Precision	Recall	F-score
Bayesian network	0.93	0.69	0.79	0.93	0.69	0.79	0.54	1.00	0.71
Decision tree	0.98	0.63	0.76	0.83	0.72	0.77	0.62	0.61	0.62
Random tree	0.76	0.77	0.76	0.79	0.77	0.78	0.69	0.71	0.70
Random forest	0.82	0.78	0.80	0.84	0.78	0.81	0.70	0.71	0.70
K-nearest neighbors	0.76	0.73	0.74	0.76	0.77	0.76	0.69	0.73	0.71

**Table 3:** Performance of classification on ADR types using DDI features on DDI corpus training data.

Classifier	ADR Type	Precision	Recall	F-score	Average Precision	Average Recall	Macro Average F-score
Bayesian network	Adverse effect	0.73	0.76	0.74	0.71	0.67	0.69
	Effect at molecular level	0.79	0.52	0.62			
	Effect related to pharmacokinetics	0.61	0.47	0.53			
	Drug interaction without known ADR	0.72	0.70	0.71			
Decision treeRandom tree	Adverse effect	0.82	0.95	0.88	0.87	0.86	0.86
	Effect at molecular level	0.87	0.85	0.86			
	Effect related to pharmacokinetics	0.82	0.77	0.79			
	Drug interaction without known ADR	0.92	0.88	0.90			
	Adverse effect	0.83	0.94	0.88	0.87	0.85	0.86
	Effect at molecular level	0.86	0.85	0.85			
	Effect related to pharmacokinetics	0.81	0.77	0.79			
	Drug interaction without known ADR	0.93	0.85	0.89			
Random forest	Adverse effect	0.84	0.95	0.89	0.88	0.86	0.87
	Effect at molecular level	0.88	0.86	0.87			
	Effect related to pharmacokinetics	0.84	0.78	0.81			
	Drug interaction without known ADR	0.94	0.86	0.90			
K-nearest neighbors	Adverse effect	0.83	0.95	0.88	0.87	0.85	0.86
	Effect at molecular level	0.86	0.84	0.85			
	Effect related to pharmacokinetics	0.81	0.76	0.79			
	Drug interaction without known ADR	0.93	0.85	0.89

**Table 4:** Performance of classification on ADR types using DDI and DGI features on DDI corpus training data.

Classifier	ADR Type	Precision	Recall	F-score	Average Precision	Average Recall	Macro Average F-score
Bayesian network	Adverse effect	0.76	0.83	0.79	0.75	0.71	0.73
	Effect at molecular level	0.83	0.59	0.69			
	Effect related to pharmacokinetics	0.67	0.47	0.56			
	Drug interaction without known ADR	0.74	0.72	0.73			
Decision tree	Adverse effect	0.85	0.96	0.90	0.89	0.88	0.89
	Effect at molecular level	0.94	0.87	0.90			
	Effect related to pharmacokinetics	0.85	0.81	0.83			
	Drug interaction without known ADR	0.91	0.92	0.91			
Random tree	Adverse effect	0.86	0.94	0.90	0.88	0.88	0.88
	Effect at molecular level	0.91	0.88	0.90			
	Effect related to pharmacokinetics	0.83	0.79	0.81			
	Drug interaction without known ADR	0.91	0.90	0.91			
Random forest	Adverse effect	0.87	0.95	0.91	0.90	0.89	0.90
	Effect at molecular level	0.93	0.89	0.91			
	Effect related to pharmacokinetics	0.86	0.82	0.84			
	Drug interaction without known ADR	0.92	0.91	0.92			
K-nearest neighbors	Adverse effect	0.86	0.94	0.90	0.89	0.88	0.88
	Effect at molecular level	0.91	0.89	0.90			
	Effect related to pharmacokinetics	0.83	0.79	0.81			
	Drug interaction without known ADR	0.91	0.90	0.91			

We compared the performance of our approach on predicting DDIs and ADR types with the top three systems namely FBK-irst system^15^, WBI system^16^ and Uturku system^17^ from DDI Extraction Shared Task 2013 on the DDI corpus test data^9^. Our approach achieved 0.831 F-score on predicting DDIs and 0.798 F-score on predicting ADR types using DDI and DGI features. We illustrated that our approach can obtain better performance than others competing methods (Supplementary Table S1).

### ADR predictions for cutaneous diseases

**Figure 2:**
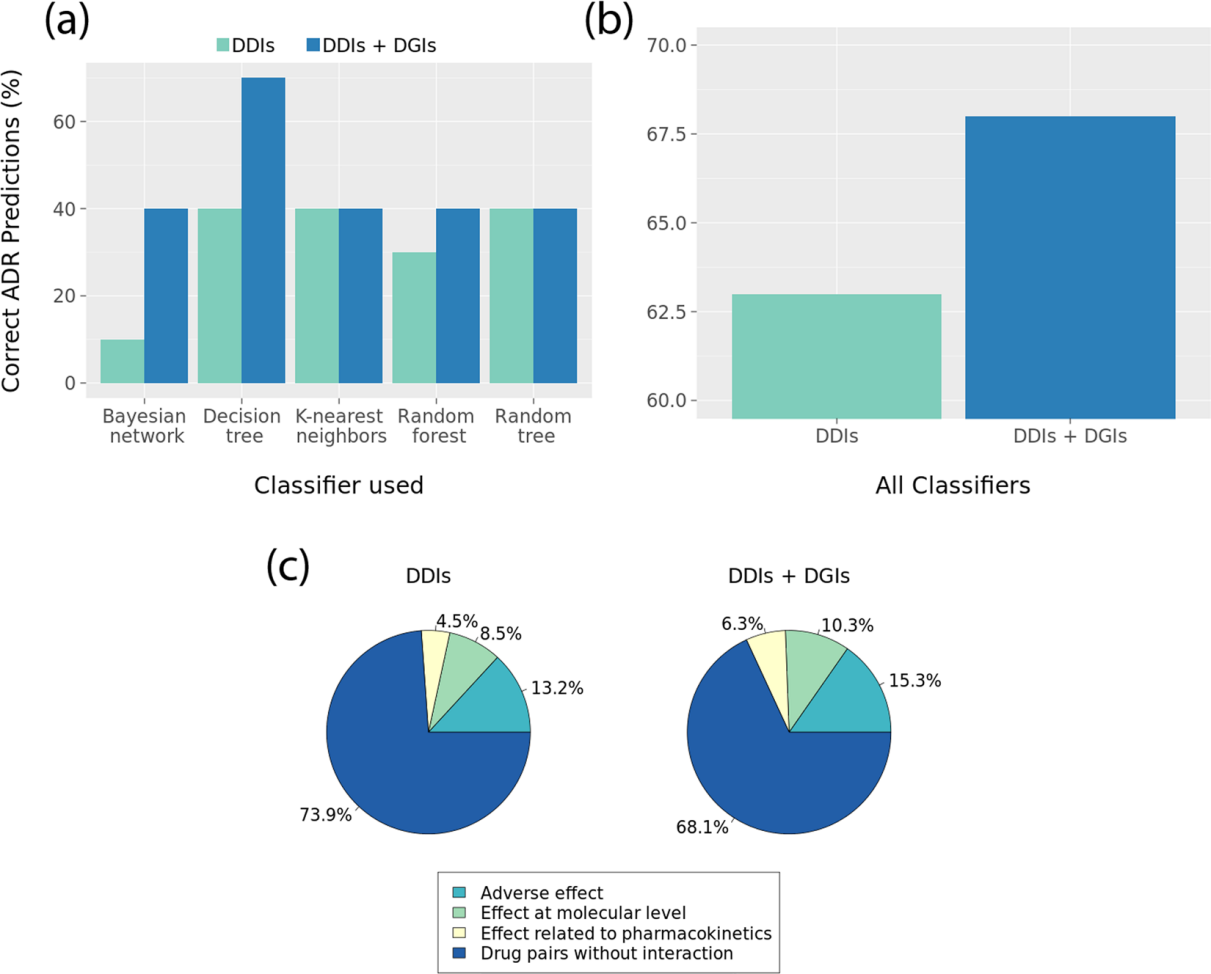
(**a**) Performance of classifiers to predict DDIs and ADR types; (**b**) Prediction of DDI and ADR types at least by three classifiers; (**c**) Performance of random forest classifier to predict DDIs and ADR types between NDFRT drugs suggested for cutaneous diseases and drugs using DDI features alone and DDI with DGI features.

We applied the machine learning workflow on 13,435 MedLine sentences that are annotated with two or more chemicals/drugs, with at least one drug for nine cutaneous diseases under study (see Methods). Among the 31,697 chemicals/drug pairs from the 13,435 sentences, only 2,646 chemicals/drug pairs associate with common genes and relationship information. We used the two training models (i.e. DDI features alone or DDI + DGI features) to predict DDIs and ADR types. We estimated the ADR predictions of each classifier as a sum of ratio of each ADR prediction to the total ADR predictions for DDI features alone or DDI + DGI features. Three out of five classifiers i.e. Bayesian network, decision tree and random forest showed improved performance with DDI + DGI features when compared to DDI features alone; with the other two classifiers perform similarly when using the two types of features (Fig. 2a). Nevertheless, the accuracy of each classifier on predicting each ADR types using DDI features alone and DDI + DGI features shows that the performance is almost equal (Supplementary Table S2). We also evaluated the accuracy of DDI and ADR predictions across the classifiers by counting the predictions achieved by at least three classifiers; interestingly the results illustrate consistently enhanced ADR predictions when using DDI + DGI features (68%) when comparing against using DDI features alone (63%) (Fig. 2b).

### Case study: ADR predictions related to psoriasis

**Figure 3:**
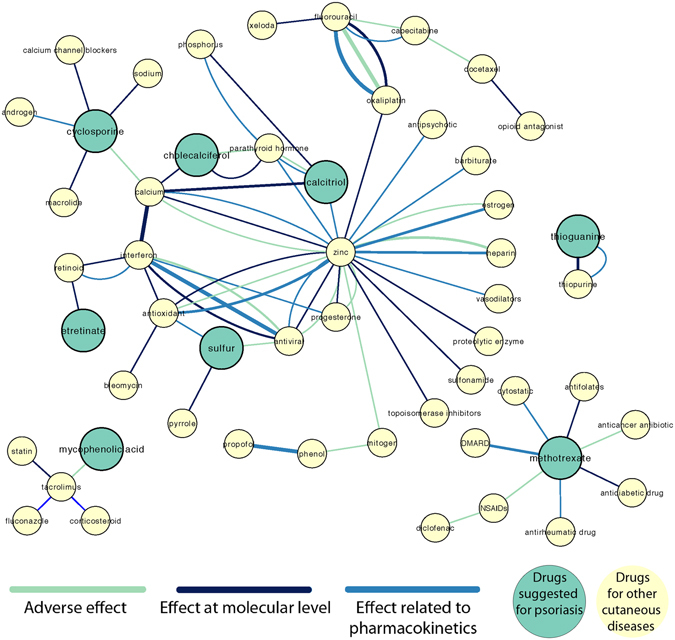
ADR network for cutaneous diseases showing interaction between NDFRT drugs suggested for cutaneous diseases and drugs. Thickness of the edges correlate with the number of instances to support the ADR predictions.

Among the nine cutaneous diseases, we chose psoriasis as a disease model to explore the underlying information that might be useful to prevent DDIs and ADRs. Psoriasis is among the fifteen diseases identified to pose significant socioeconomic and public health burden^18^. In the United States, the cost spent on psoriasis is estimated to be approximately $112 billion dollars annually^19^. We believe that information on DDI and ADR types for psoriasis could help in deciding more efficient treatment, thus reduce the burden. We used random forest classifier to perform the analysis, as it shows the best performance on classifying DDIs and ADRs on the DDI corpus. The classifier predicted a total of 3,109 DDIs and ADR types (i.e. 1,335 DDIs for adverse effect; 995 DDIs for effect at molecular level; 766 DDIs for effect related to pharmacokinetics; and 13 DDIs for DDIs without known ADRs). Manual inspection on 3,109 DDIs revealed interaction with chemicals (e.g. copper). Since such information might not be useful in clinical perspectives, we excluded the interactions involving chemicals. The approach yielded 177 DDIs and ADR types: 85 DDIs for adverse effect (15.3%); 57 DDIs for effect at molecular level (10.3%); and 35 DDIs for effect related to pharmacokinetics (6.3%) (Fig. 2c). Among these, eight drugs (out of 32 drugs) suggested for psoriasis treatment (i.e. methotrexate, etretinate, thioguanine, cyclosporine, cholecalciferol, calcitriol, mycophenolic acid and sulfur) were predicted to have interaction with other drugs. Figure 3 shows the DDIs and ADR types predicted for psoriasis. In addition, we tested the robustness of the machine learning workflow by tracing random samples of the predicted DDIs back to PubMed sentences, and confirmed accurate predictions. Supplementary Data S1 lists PubMed sentences with DDI and ADR information that were successfully predicted by the machine learning workflow.

#### Genes in ADR prediction

**Figure 4:**
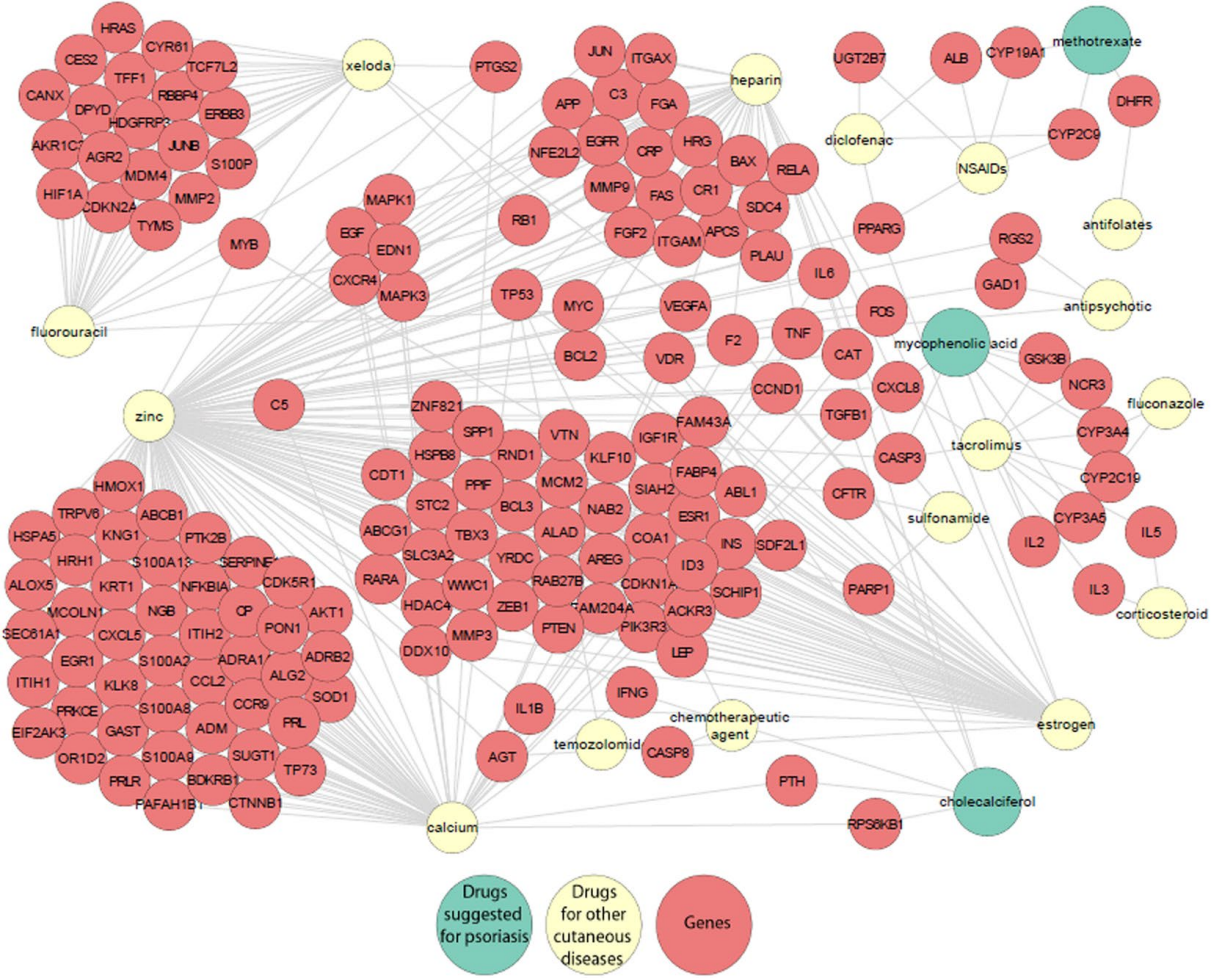
Gene - DDI network for cutaneous diseases showing interaction between NDFRT drugs suggested for cutaneous diseases/drugs with genes.

Among the 177 ADRs predicted for psoriasis medications, 31 DDIs are associated with common genes (11 for adverse effect; 10 for effect at molecular level; and 10 for effect related to pharmacokinetics). Among these, three drugs methotrexate, cholecalciferol and mycophenolic acid are suggested for psoriasis. Therefore, by incorporating the DGI information, our approach can predict DDIs and ADRs if the drug pairs are not present in the same sentence from the literature. We believe that our predictions could be useful to decide the right treatment for patients with psoriasis or other cutaneous diseases (Fig. 4).

#### Diseases related to ADR prediction

Our population is aging, and it is getting more common to prescribe multiple drugs for complex mulimorbidity^20^. Knowledge on DDI and ADRs would be useful for medical assessment especially for diseases that co-occur in higher rate (i.e. comorbidity). We performed a study to identify the diseases associated with DDI pairs from various sources such as NDFRT, DrugBank, UpToDate, CDC, and Mayo clinic’s Diseases and Conditions (Supplementary Table S3). For psoriasis we identified the following interesting disease comorbidity through DDIs: Crohn’s disease, rickets in children, osteomalacia in adults, breast cancer in females, lupus erythematosus, eczema, hypocalcemia, diabetes, atopic dermatitis, blood pressure, influenza, Raynaud’s disease, melanoma and bacterial conjuctivities.

## Discussion

In this study we assessed the feasibility of predicting DDIs and ADR types using machine learning workflow, and evaluated the performance by incorporating DGI information and compared that with using DDI information alone. We applied the workflow to predict DDIs and classified DDIs to ADR types related to cutaneous diseases, and conducted an intense analysis on DDIs and ADR types related to psoriasis. We extended the finding to identify comorbid diseases related to cutaneous diseases.

Certain drugs used for treating the cutaneous diseases are chemicals. Therefore, our lexicon includes all UMLS TUIs belonging to Chemicals and Drugs. However, the annotations in DDI corpus include only drugs. We conducted a second experiment to remove the chemical entities and identified the performance of the lexicon. To achieve this, we considered the entities belonging to three TUIs i.e. clinical drugs, antibiotics and pharmacologic substances as drugs. We incorporated the lexicon with MedTagger^21^ a widely used tool for concept extraction. The performance achieved by the lexicon on DDI corpus is impressive and we believe that the concept extraction related to cutaneous diseases is equally good. Our approach to predict DDIs and ADR types was carried out in two steps: to classify the sentences with potential DDIs and to classify DDIs to ADRs. We tested both the steps with different classifiers: Bayesian network, logistic regression, support vector machines, decision tree, random tree, random forest, k-nearest neighbors and multilayer perceptron.

Among 31,268 annotated drug pairs from 3,788 sentences in the DDI corpus, only 5,773 drug pairs from 1,441 sentences contain DGI information. Our attempt to compare DDI classification only on 5,773 drugs pairs achieved an increase of 3–6% F-score with DDI + DGI features when compared to DDI features alone (Supplementary Table S4). In addition, we analyzed the predictions by random forest classifier on 10-fold cross validation using DDI features alone and DDI + DGI features. We observed that the average false positive rate between DDI features alone and DDI + DGI features decreased by ~23% (i.e. 0.448 to 0.220) on predicting true DDIs and ~3% (i.e. 0.249 to 0.220) on classifying drug pairs without interaction. We believe that this decrease in average false positive rate is significantly important in the clinical perspectives of applying our approach to predict DDIs and ADR types. We also compared DDI features alone and DDI + DGI features on four statistical error measures namely mean absolute error, root mean squared error, relative absolute error and root relative squared error. A decrease in error between DDI features alone and DDI + DGI features by all statistical error measures further confirms the enhanced performance of DDI_DGI features than DDI features alone (Supplementary Table S5).

Among the selected DDI and DGI features, the mean impurity decrease were used to evaluate the importance of each feature (Supplementary Table S6). We also observed that the features are unique to a specific ADR type and thus can facilitate its prediction. For example, the most significant features such as “patients” and “absorption” are highly specific to “adverse effects” ADR type; the features such as “increase” and “effect” are highly specific to “effect at molecular levels” ADR types; and the feature “metabolism” is highly specific to “effects related to pharmacokinetics”. The negated features (e.g. ‘no effect’) are important to identify drug pairs without interaction, and our regression model is capable of identifying the negated features as significant ones.

We evaluated the performance of DGI features alone on predicting DDIs and ADR types. The approach showed a decrease of 3–14% F-score when compared to DDI features alone and 5–15% F-score when compared to DDI + DGI features by different classifiers (Table 2). The performance of DGI features alone on ADR classification showed a decrease of 14–26% precision when compared to DDI features alone and 18–28% precision when compared to DDI + DGI features by different classifiers. The recall achieved by all the classifiers is very low (i.e. 0.25 to 0.26) because, the gene association information is available only for 5,773 drug pairs (out of 31,268 annotated drug pairs) in the DDI Corpus (Supplementary Table S7).

## Conclusion

A comprehensive knowledge regarding potential ADRs is essential to provide better treatment. It is thus imminent to have efficient approach to predict adverse drug-drug interactions as a screening procedure for subsequent validations. Aiming to provide an automated approach to predict in advance medication-related to DDIs and ADR types prediction, we present here a workflow that integrates machine learning approach with biomedical literature data to data-mine potential drug-drug interactions for cutaneous diseases. We evaluated our approach on DDI corpus using DDI features alone, and DDI + DGI features. We also showed that the performance of machine learning workflow improved when DGI is incorporated with DDI. We then applied this approach to predict chemicals/drugs that interact with prescribed drugs for cutaneous diseases. In the current study we advance the earlier approach on inferring DDI through DGI by classifying the extracted DDIs to their specific ADR types.

## Methods

### Overview

Our approach for predicting DDI-based ADR types (Fig. 1) consists of the following steps: (*1*) building a lexicon for chemicals and drugs (*L*); (*2*) mapping *L* to the DDI corpus (for training) and MedLine abstracts (for application); (*3*) extracting drug/chemical to gene interactions from the Comparative Toxicogenomics Database (CTD)^13^; (*4*) using machine learning approaches to classify literature sentences for DDIs and then categorize different ADR types.

### Chemicals and drugs lexicon

The chemicals and drugs lexicon was compiled from three resources: UMLS Metathesaurus^22^, DrugBank^23^ and PharmGKB^24^. The 2015AB version of UMLS Metathesaurus was downloaded and customized to Rich Release Format (RRF) using MetamorphoSys, an UMLS installation wizard and Metathesaurus customization tool. UMLS Metathesaurus contains more than 3.2 million health related concepts and 12.8 million unique concepts from over 190 source vocabularies (e.g. SNOMED, ICD 9/10). We selected only concepts belonging to semantic groups “Chemicals and Drugs” in subsequent analysis. UMLS concepts are assigned with one or more semantic types (TUI) to define the category (e.g. Clinical Drugs, Antibiotics). We conducted a screening approach to remove the concepts appearing in multiple semantic groups, common English terms as brand names, and abbreviations to minimize mapping errors. Since UMLS Metathesaurus does not cover all possible synonyms for a concept^25^, we expanded the lexicon to the synonyms used in the common drug databases: DrugBank^23^ and PharmGKB^24^. DrugBank contains 8,203 drugs and 1,201 drug salts; and PharmGKB contains information for 3,175 drugs. Since certain drugs and synonyms contain Greek alphabets (e.g. α-methylthiofentanyl) and other Unicode characters (e.g. R-N[2-[1-(aminoiminomethyl)-3-piperidinyl]-1-oxoethyl]-4-(phenylethynyl)-l-phenylalanine methylester), we used UTF-8 encoding to extract the drug names.

### DDI corpus

The “drug-drug interaction (DDI) corpus” is a major contribution of DDI Extraction Shared Task 2013^9^, in which two domain experts and two miners manually annotated 6,793 DrugBank documents and 1,701 MedLine documents (Supplementary Table S8). Each document includes up to 300 binary DDI annotations (i.e. “True”/“False” between any two drugs), and the “True” annotations include four DDI-derived ADR types: *i*) adverse effect; *ii*) effect at molecular level; *iii*) effect related to pharmacokinetics; *iv*) and drug interactions without known ADR.

### MedLine abstracts

For applying our approach to infer DDIs and ADRs for drugs of cutaneous diseases, we retrieved the most recent version of MedLine citations. The 2015 MedLine baseline XML files were downloaded from the ftp server maintained by the National Library of Medicine (NLM) (https://www.nlm.nih.gov/). We restricted our study to the 482,380 filtered citations with abstracts and annotated with human genes based on the gene2pubmed file from NCBI. In addition, we only utilized 469,995 (i.e. >97%) citations that map to at most five human genes to maintain precision in gene-mapping for the subsequent analysis **(**Supplementary Table S9 **)**. We used a recently developed rule-based approach^26^ to conduct abbreviation expansion and sentence segmentation. Briefly, we obtained a range of words preceding the abbreviation that could contain the original term; we then extracted the original term by using two constraints: (*i*) the order of character matching must not change; and (*ii*) the first character of the original term and the abbreviation must match. The segmented sentences were assigned with PMIDs. The process yielded a set of 4,712,812 sentences from 469,995 MedLine abstracts.

### Extraction of Drugs/Chemicals

Identification of ADRs derived from DDIs requires name recognition of drugs/chemicals as well as extraction of the potential interactions between the drugs. Chemicals and drugs can contain more than one words (e.g. ACE inhibitors). We used MedTagger^21^ to map Drugs/Chemicals from the DDI corpus and MedLine sentences. MedTagger is an extraction pipeline that consists of tokenization, lexical normalization, dictionary look-up, and screening. We used the constructed lexicon as the concept dictionary for MedTagger to extract chemicals/drugs from the DDI Corpus and MedLine sentences.

### Extraction of Chemical/Drug-Gene interaction

DDIs may occur when two or more drugs interact with the same gene^8^. We hypothesized that information regarding DGIs can enhance the prediction of DDIs as well as ADR types classification by using machine-learning approaches. While databases such as Comparative Toxicogenomics Database (CTD)^13^ or Drug-Gene Interaction Database (DGIdb)^27^ provide valuable resources regarding DGIs and their association types (e.g. acetylation, methylation, phosphorylation), to our knowledge these resources have not been used to predict potential DDIs and associated ADR types in common practice. We retrieved >500,000 DGIs from CTD, pertaining to 21,986 human genes and 8,176 chemicals/drugs from 24,311 MedLine articles. As the identifiers in CTD might not be identical to those in our compiled lexicon, we used the synonyms from our lexicon to map DGIs in CTD. For each drug pair, we searched for gene(s) from the CTD database that interacts with both the drugs and retrieved the DGI associations. The process obtained 193,294 DGIs for 5,773 drug pairs (out of 31,268 annotated drug pairs) in DDI Corpus, and 49,188 DGIs for 935 drug pairs (i.e. at least one drug in each pair is prescribed as a medication for cutaneous diseases) in MedLine sentences.

### Classification

#### DDI-based feature selection

We selected 2,893 documents from DDI corpus with at least one DDI annotation. Feature selection process was employed to identify the DDI-based features and DGI-based features. The approach for selecting DDI-based features consists of three steps: (1) collecting list of non-frequent vocabularies; (2) generating frequency matrices by mapping vocabularies to each document; and (3) selecting features using stepwise logistic regression model. First, we preprocessed the documents to remove the stop-words, chemical/drug mentions and numbers (e.g. “40.8”, “ >80”, “75.3%”). The process retrieved 2,101 non-frequent vocabularies. We computed two measures for each vocabulary: the term frequency-inverse document frequency (TF-IDF) and negation vocabulary phrase (NVP) metrics. For each document, we generated a parse tree structure using the Stanford lexical parser and applied the Tregex syntactic pattern matcher to identify the negation meanings (e.g. no pharmacokinetic activity) in each vocabulary within the document. Any vocabulary appearing within the same noun/verb phrases of the negation keywords (i.e. no, not, without, neither, nor, cannot) is considered as NVP, and we assigned binary (1/0) values for each vocabulary; we assigned zero TF-IDF score for any vocabulary within NVP. The TF-IDF and NVP measures were used to indicate the importance of a vocabulary in the corpus collection, as well as to indicate the presence of negation meaning. For all the vocabularies in the TF-IDF matrix, we used a stepwise heuristic algorithm to identify the top nominal significant (*p* < *0*.*05*) features using logistic regression framework. We identified and employed the top 24 significant DDI-based features in subsequent analysis. In addition, we also included in the classification the following features: for each sentence, the total number of words, drugs, and features; and the minimum number of features present before, between, after each drug pair. The generated feature vector consists of the features and the class label (True/False).

#### DGI-based feature selection

We generated two types of models, one having DDI features alone and the other having DDI and DGI features. The approach for selecting DGI-based features consists of mapping drug names, genes, and drug-gene associations from CTD. For each drug pair, we identified a set of interacting genes common to both of the drugs, as well as their drug-gene associations from CTD. We applied the stepwise heuristic algorithm described above to identify the top significant features. The process yielded 20 top significant DGI-based features.

#### Classifying DDIs and ADR types

We used machine learning approaches implemented in Weka (3.6.0)^28^ to classify DDIs and ADR types by utilizing five different types of classifiers: Bayesian Network, Decision tree, Random tree, Random forest, and K-nearest neighbors. Ten-fold cross validation was used for evaluation. We examined and compared the performance when using the DDIs or the DDIs + DGIs features identified above for each classification task. The four types of ADR classes declared for the classification are: *i*) adverse effect; *ii*) effects at molecular level; *iii*) effects related to pharmacokinetics; and *iv*) drug interactions without known ADRs. We also allowed the classifier to classify DDIs that do not fall under the four categories. The training data includes 3,767 true ADR (i.e. all four types) annotations and 22,216 false ADR annotations. The distribution of true ADR vs. false ADR is imbalanced, and this may result in performance degradation of classifiers because the predictions more tend to fall under the majority class. Therefore, we applied Synthetic Minority Oversampling Technique (SMOTE) for class balance^29^. SMOTE is one of the most well-known data preprocessing techniques that achieves class balance by over-sampling the minority class (i.e. all four types of true ADRs) and under-sampling the majority class (i.e. false ADRs).

We performed ADR types classification for each drug pair from the document that was classified as “true” in the DDI classification. We used the features identified by the above procedure to classify ADR types. In addition, we included the count of drugs between the drug pairs for the ADR type classification as feature.

### Application on cutaneous diseases

ADRs are frequent events that affect up to 3% of hospitalized patients^30^, and skin reactions can be potentially induced by most pharmacologic agents^31–34^, thus suggesting how skin as ground tissue can elicit the adverse effect related to pharmacokinetics (i.e. Absorption, Distribution, Metabolism and Elimination) manifested^30^. Therefore, we applied our approach to identify medications for cutaneous diseases that might induce adverse reactions when taken together with other drugs. Medications for cutaneous diseases include topical or oral treatments, and they target a variety of purposes, including immunosuppresants, fungal/bacterial infection, and enzyme inhibitors. The relationship between disease and drug is available at NDFRT^14^. In this study, we extracted a list of 197 unique drugs from NDFRT for the following nine cutaneous diseases: psoriasis, atopic dermatitis, rosacea, acne vulgaris, alopecia, melanoma, eczema, keratosis, and pruritus (Supplementary Table S10). We mapped these drugs to 4,712,812 MedLine sentences (Fig. 1). The process extracted 13,435 MedLine sentences mapped with 171 drugs (Supplementary Table S11), and we used 13,435 sentences to identify sentences with potential DDIs and their associated ADR types. Similar to drug pairs in DDI corpus, we mapped the DGI information for 197 drugs. Only 70 out of 197 drugs contain DGI information. We used both the training models i.e. DDI features only, and combined DDI and DGI features for classifying sentences with potential DDIs and ADRs.

### Evaluation

We adapted the standard evaluation metrics of precision, recall, and F-score to measure the system performance on *i*) identifying chemical and drug names; *ii*) classifying sentences with potential DDI; and *iii*) classifying DDI into four ADR types. We used the annotations from DDI corpus as a gold standard. For measuring the performance on identifying chemicals and drugs, we defined TP (true positive) as the number of chemicals and drugs identified correctly, FP as the number of chemicals and drugs identified incorrectly, and FN as the number of chemicals and drugs not identified by the system. The partially identified chemical or drug names (i.e. only part of the names were identified) are rated as FN. For measuring the performance on classifying DDI, we define TP as the number of DDIs correctly classified as true or false, FP as the number of DDIs classified incorrectly, and FN as the number of DDIs not classified by the system. The performance measure for classifying the ADR types is adapted from the evaluation metrics used in the DDI Extraction Shared Task 2013^9^. There are five output types i.e. four ADR types and a false type for drug pairs that do not fall into any ADR types. We also calculated the macro-averaged F-score from micro average precision and recall of all ADR types.

## Electronic supplementary material

Supplementary Info
