# Supplementary material for: Machine learning workflow to enhance predictions of Adverse Drug Reactions (ADRs) through drug-gene interactions: application to drugs for cutaneous diseases
Source: Sci Rep. 2017 Jun 16;7:3690. doi: 10.1038/s41598-017-03914-3 (PMC5473874; doi:10.1038/s41598-017-03914-3)
Supplement: Supplementary file 1 — Supplementary Info [file 41598_2017_3914_MOESM1_ESM.pdf]

Machine learning workflow to enhance predictions of Adverse Drug Reactions (ADRs) through drug-gene interactions: application to drugs for cutaneous diseases

Kalpana Raja[1], Matthew Patrick[1], James T Elder[1], Lam C Tsoi[1,2,3*]

Supplementary Table S1. Performance comparison with the existing systems on DDI corpus test data

System	Description	Classifier	DDI classification			ADR categorization		
			**P**	**R**	**F**	**P**	**R**	**F**
Our approach	DDI features	Random forest	0.739	0.823	0.779	0.761	0.793	0.755
	DDI + DGI features		0.875	0.790	0.831	0.839	0.761	0.798
FBK-irst system	Contextual and shallow linguistic features	Support vector machines	0.794	0.806	0.800	0.633	0.642	0.638
WBI system	Ensembles of five different classifiers	Shallow linguistic kernel + a self-developed feature based classifier + Turku event extraction system	0.801	0.722	0.759	0.642	0.579	0.609
Uturku system	Deep syntactic features and information from external domain resources	Turku event extraction system	0.833	0.602	0.699	0.732	0.499	0.594

Supplementary Table S2. ADR predictions on MedLine abstracts related to cutaneous diseases

	Adverse Effect (%)		Effect at molecular level (%)		Effect related to pharmacokinetics (%)		DDIs without known ADRs (%)		False ADRs (%)	
	**DDI**	**DDI and DGI**	**DDI**	**DDI and DGI**	**DDI**	**DDI and DGI**	**DDI**	**DDI and DGI**	**DDI**	**DDI and DGI**
Bayesian Network	4.7	3.1	2.3	2.8	1.7	1.8	2.0	2.5	89.1	90.6
Decision Tree	13.8	18.3	14.6	31.9	15.3	15.1	0.0	5.3	56.2	58.2
Random Tree	15.0	14.8	12.3	4.1	10.7	13.7	0.0	5.3	63.6	62.1
Random Forest	14.0	14.2	10.7	4.1	8.7	13.5	0.0	5.3	66.5	63.0
K-nearest neighbors	18.0	15.8	10.3	4.1	10.6	13.9	0.0	5.3	63.8	61.0

Supplementary Table S3. Cutaneous diseases and their comorbid diseases identified through ADRs

Drug1	Drug2	Disease for Drug1	Disease for Drug2
Cyclosporine	Calcium	Psoriasis	Bone related diseases
Methotrexate	Anticancer antibiotic	Psoriasis	Cancer
Thioguanine	Thiopurine	Psoriasis	Acute lymphoblastic leukemia|autoimmune disorders (Crohn's disease, rheumatoid arthritis)
Calcitriol	Calcium	Psoriasis	Bone related diseases
Calcitriol	Phosphorus	Psoriasis	Bone related diseases - Rickets in children, Osteomalacia in adults
Methotrexate	DMARD	Psoriasis	Rheumatoid arthritis, Lupus erythematosus, Psoriasis
Calcitriol	Zinc	Psoriasis	Eczema
Sulfur	Antioxidant	Acne vulgaris, Psoriasis, Rosacea	Cancer
Mycophenolic acid	Tacrolimus	Psoriasis	Atopic dermatitis
Cyclosporine	Androgen	Psoriasis	Breast cancer in females
Cholecalciferol	Parathyroid hormone	Psoriasis	To control hypocalcemia in patients in hypoparathyroidism
Calcitriol	Parathyroid hormone	Psoriasis	To control hypocalcemia in patients in hypoparathyroidism
Cyclosporine	Sodium	Psoriasis	Blood pressure and blood volume
Cholecalciferol	Calcium	Psoriasis	Bone related diseases
Methotrexate	Antidiabetic drug	Psoriasis	Diabetes
Sulfur	Antiviral	Acne vulgaris, Psoriasis, Rosacea	Viral diseases - Influenza (flu)
Methotrexate	Antirheumatic drug	Psoriasis	Rheumatoid arthritis
Cyclosporine	Calcium channel blockers	Psoriasis	High blood pressure, Chest pain, Raynaud's disease
Methotrexate	Antifolates	Psoriasis	Cancer

Methotrexate	NSAIDs	Psoriasis	Fever, pain, inflammation
Cyclosporine	Macrolide	Psoriasis	Bacterial conjuctivities
Etretinate	Retinoid	Psoriasis	Melanoma
Diclofenac	NSAIDs	Keratosis	Fever, pain, inflammation
Fluorouracil	Xeloda	Keratosis	Colorectal neoplasms
Tacrolimus	Corticosteroid	Atopic dermatitis	Rheumatoid arthritis, Lupus, Asthma, Allergies, Addison's disease
Tacrolimus	Fluconazole	Atopic dermatitis	Cryptococcal meningitis, AIDS-related opportunistic infections, Fungemia, Vulvovaginal candidiasis, Histoplasmosis, Chronic mucocutaneous candidiasis, Histoplasmosis, Coccidioidomycosis, Blastomycosis
Temozolomide	Chemotherapeutic agent	Melanoma	Cancer
Zinc	Heparin	Eczema	Thromboembolism, Thrombophlebitis, Pulmonary embolism, unstable Angina, Myocardial infarction, Cerebral infarction, postoperative complications, Coronary thrombosis
Zinc	Progesterone	Eczema	Endometrial hyperplasia, Uterine hemorrhage, female infertility, Amenorrhea
Zinc	Calcium	Eczema	Bone related diseases
Zinc	Estrogen	Eczema	Menorrhagia, breast neoplasms, premature menopause, primary ovarian insufficiency, Hypogonadism, Prostatic neoplasms, hot flashes
Zinc	Antipsychotic	Eczema	Schizoprenia
Zinc	Sulfonamide	Eczema	Acne vulgaris, Acne rosacea, Seborrheic dermatitis

Supplementary Table S4. DDI features vs. DDI with DGI features on drug pairs with gene association information

**Classifier Model**	**DDI features**			**DDI with DGI feature**		
	**P**	**R**	**F**	**P**	**R**	**F**
Baysian Network	0.818	0.622	0.707	0.833	0.688	0.754
J48	0.818	0.608	0.697	0.952	0.642	0.767
Random Tree	0.724	0.741	0.733	0.768	0.793	0.781
Random Forest	0.753	0.754	0.753	0.832	0.780	0.805
K nearest neighbors	0.725	0.741	0.733	0.756	0.769	0.763

Supplementary Table S5. Statistical error measures for DDI features only vs. DDI+DGI features

Statistical error measure	DDI features	DDI+DGI features
Mean absolute error	0.3824	0.2610
Root mean squared error	0.4723	0.3739
Relative absolute error	0.7867	0.5370
Root relative squared error	0.9582	0.7584

Supplementary Table S6. Effect of features on DDI classification and ADR categorization

		Stepwise logistic regression model (p-value)	Mean impurity decrease			
			DDI classification		ADR categorization	
			DDI Features	DDI+DGI Features	DDI Features	DDI+DGI Features
**DDI Features**	increase	6.57e-14	0.24	0.21	0.12	0.14
	effect (as negation)	5.86e-12	0.21	0.17	0.00	0.00
	patients	8.38e-11	0.10	0.06	0.19	0.22
	decrease	3.57e-08	0.28	0.24	0.11	0.11
	absorption	5.90e-07	0.13	0.11	0.12	0.12
	decreased	1.15e-07	0.05	0.19	0.10	0.12
	levels	1.86e-06	0.20	0.14	0.19	0.20
	auc	7.95e-06	0.16	0.19	0.08	0.08
	effects	2.92e-05	0.10	0.06	0.16	0.16
	metabolism	1.65e-05	0.18	0.11	0.15	0.15
	administration	1.31e-05	0.19	0.20	0.15	0.15
	enhance	3.34e-05	0.07	0.15	0.09	0.11
	significantly (as negation)	4.61e-05	0.13	0.07	0.00	0.00
	inhibited	0.0201	0.12	0.14	0.04	0.05
	increasing	0.0046	0.12	0.13	0.02	0.30
	antihypertensive	0.0021	0.19	0.20	0.05	0.04
	alter (as negation)	0.0014	0.17	0.10	0.00	0.00
	pressure	0.0010	0.06	0.13	0.00	0.00
	approximately	0.0009	0.15	0.13	0.24	0.25
	potentiate	0.0005	0.18	0.16	0.00	0.00
	resulted	0.0004	0.16	0.02	0.05	0.08
	monitored	0.0003	0.15	0.19	0.11	0.11
	administered	0.0001	0.16	0.11	0.18	0.16
	clearance	0.0001	0.15	0.20	0.11	0.13
**Additional features**	total words between drug pairs	-	0.20	0.43	0.31	0.33
	total drugs between drug pairs	-	0.22	0.35	0.18	0.20

	minimum number of features preceding drug pairs	-	0.26	0.23	-	-
	minimum number of features between drug pairs	-	0.30	0.21	-	-
	minimum number of features succeeding drug pairs	-	0.31	0.26	-	-
**DGI Features**	acetylation : glutathionylation	-	-	0.30	-	0.22
	chemical synthesis : hydrolysis	-	-	0.07	-	0.21
	expression : hydroxylation	-	-	0.25	-	0.14
	expression : glucuronidation	-	-	0.17	-	0.14
	activity : oxidation	-	-	0.18	-	0.14
	binding : response to substance	-	-	0.24	-	0.12
	hydroxylation : hydroxylation	-	-	0.20	-	0.11
	oxidation : response to substance	-	-	0.20	-	0.09
	activity : chemical synthesis	-	-	0.21	-	0.09
	expression : splicing	-	-	0.08	-	0.09
	expression : stability	-	-	0.15	-	0.07
	acetylation : response to substance	-	-	0.15	-	0.07
	import : transport	-	-	0.12	-	0.06
	glutathionylation : response to substance	-	-	0.12	-	0.06
	degradation : methylation	-	-	0.11	-	0.06
	localization : phosphorylation	-	-	0.16	-	0.03
	binding : methylation	-	-	0.09	-	0.03
	activity : mutagenesis	-	-	0.06	-	0.03
	sulfation : sulfation	-	-	0.15	-	0.02
	oxidation : oxidation	-	-	0.12	-	0.02

Supplementary Table S7. Performance of various classifiers using DGI features only

Classifier	ADR Type	Precision	Recall	F-score	Average Precision	Average Recall	Macro Average F-score
Bayesian network	Adverse effect	0.62	0.10	0.16	0.57	0.25	0.34
	Effect at molecular level	0.67	0.06	0.11			
	Effect related to pharmacokinetics	0.42	0.07	0.12			
	Drug interaction without known ADR	0.88	0.07	0.12			
Decision tree	Adverse effect	0.64	0.10	0.17	0.61	0.25	0.36
	Effect at molecular level	0.60	0.08	0.14			
	Effect related to pharmacokinetics	0.66	0.05	0.10			
	Drug interaction without known ADR	0.85	0.08	0.14			
Random tree	Adverse effect	0.64	0.10	0.17	0.62	0.26	0.36
	Effect at molecular level	0.60	0.09	0.15			
	Effect related to pharmacokinetics	0.67	0.06	0.10			
	Drug interaction without known ADR	0.87	0.07	0.13			
Random forest	Adverse effect	0.65	0.10	0.18	0.62	0.26	0.36
	Effect at molecular level	0.60	0.09	0.16			
	Effect related to pharmacokinetics	0.67	0.06	0.11			
	Drug interaction without known ADR	0.87	0.07	0.13			
K-nearest neighbors	Adverse effect	0.65	0.10	0.17	0.61	0.25	0.36
	Effect at molecular level	0.60	0.09	0.16			
	Effect related to pharmacokinetics	0.66	0.05	0.10			
	Drug interaction without known ADR	0.87	0.07	0.13			

Supplementary Table S8. Documents and annotations in DDI Corpus

DDI Corpus	XML Files		Documents		Annotations		
	**DrugBank**	**MedLine**	**DrugBank**	**MedLine**	**Type**	**DrugBank**	**MedLine**
Training data	572	142	5,675	1,301	True – Adverse effect	818	8
					True – Effect at molecular level	1,535	152
					True – Effect related to pharmacokinetics	1,256	62
					True – Drug interaction	178	10
					False	22,216	1,555
Test data – Named Entity Recognition Task	54	58	143	83	-	-	-
Test data – DDI extraction Task	158	33	973	326	True – Adverse effect	214	7
					True – Effect at molecular level	298	62
					True – Effect related to pharmacokinetics	278	24
					True – Drug interaction	94	2
					False	4,381	356

Supplementary Table S9. Gene distribution in MedLine articles

Number of genes	Number of PMID
1-5	469,995
6-10	8278
11-15	1639
16-20	735
21-25	386
26-30	184
31-35	193
36-40	119
41-45	88
46-50	56
51-55	39
56-60	51
61-65	42
66-70	37
71-75	30
76-80	37
81-85	23
86-90	19
91-95	23
95-100	16
>100	394

Supplementary Table S10. NDFRT drugs for skin diseases

Disease	Number of unique Drugs
Psoriasis	50
Dermatitis, Atopic	25
Rosacea	12
Acne vulgaris	58
Baldness (Alopecia)	3
Melanoma	26
Eczema	4
Keratosis	6
Pruritus	42

Supplementary Table S11. MedLine sentences mapped with NDFRT drugs

	Number of sentences	Number of MedLine abstracts
All sentences	4,712,812	469,995
Sentences with two or more chemicals / drugs	794,403	301,199
Sentences with two or more chemicals / drugs with at least one NDFRT drug	13,435	8,258

Supplementary Data S1: PubMed Sentences with ADR information, predicted by machine learning workflow. Drug names are in bold.

“Simultaneous use of nonsteroidal anti-inflammatory drugs **NSAIDs** probenecid and other drugs has been reported to delay the plasma elimination of **methotrexate** in patients”.[32]

“The decreased **parathyroid hormone** levels would then also contribute to a decrease in **calcitriol** synthesis”.[33]

“Our findings show that FKBP51 and Cyp40 are positive regulators of androgen receptor that can be selectively targeted by **cyclosporine** A and **FK506** to achieve inhibition of **androgen** induced cell proliferation”.[34]

“Albeit its great benefits as immunosuppressant, the use of **Cyclosporine** A has been limited by undesirable nephrotoxic effects, including **sodium** retention, hypertension, hyperkalemia, interstial fibrosis and progressive renal failure in transplant recipients”.[28]
